# Revision knee arthroplasty using a modular system manufactured in Brazil. Clinical and radiographic results with a mean nine-year follow-up

**DOI:** 10.1016/j.clinsp.2024.100508

**Published:** 2024-10-05

**Authors:** Francisco Fontes Cintra, Mauricio Etchebehere, Eduardo Rached, Giancarlo Cavenaghi, Paulo Eduardo Dias Rahal, Rodrigo Gonçalves Pagnano

**Affiliations:** a Universidade Estadual de Campinas, Campinas, SP, Brazil; b Pontifícia Universidade Católica de Campinas, Campinas, SP, Brazil; c Hospital Estadul de Sumaré, Sumaré, SP, Brazil

**Keywords:** Arthroplasty, Replacement, Knee

## Abstract

**Objective:**

To demonstrate the clinical and radiographic results of revision knee arthroplasty using a modular system manufactured in Brazil.

**Methods:**

Between November 2010 and January 2017, 31 revision knee arthroplasties were performed in 30 patients, using the MB-V system. Patients were assessed clinically and radiographically after a minimum follow-up of two years using the following scores: Knee Society Score (KSS), Knee Society Score ‒ Function (KSS ‒ Function), and Knee Society Roentgenographic Evaluation System (KSRES).

**Results:**

Among the patients, 19 were women. The mean age at surgery was 68 years. The mean follow-up was 9.1 years. There was no aseptic loosening in this period. The mean values of KSS, KSS-function, and KSRES were, respectively, 82, 77, and less than 4. One patient evolved with postoperative flexion instability. Two knees became infected, requiring the removal of the prosthesis and implantation of spacers loaded with antibiotics. One patient developed arthrofibrosis. One patient had a dislocation with full medial collateral avulsion and had to be re-operated with an insert exchange to a CCK liner and medial collateral reconstruction as proposed by Krakow.

**Conclusion:**

This implant had 93.5 % survivorship (no cases of aseptic failure) and good results in 27 out of 31 knees (87 % of the surgeries).

## Highlights

• MBV revision knee modular system (MBVS) could be a solution for complex scenarios.
• In a mean follow up of 9.1 years, the MBVS showed 93.5 % of overall survivorship.
• The MBV should not be used in cases with major instability.

## Introduction

Revision Knee Arthroplasty (RKA) is a difficult surgery due to poor bone stock, ligament instability, expensive cost implants, blood loss and inferior patient satisfaction (related to primary surgery).^1^, ^2^, ^3^, ^4^, ^5^^,^^7^ Revision knee implants have evolved to provide modular systems, different types of fixation methods, and increasing levels of ligament constraint.^1^, ^2^, ^3^, ^4^, ^5^, ^6^, ^7^, ^8^, ^9^ Most systems use stems to improve implant fixation. The fixation methods available are: a) Total cemented (canal and metaphyseal bone); b) Hybrid (non-porous diaphyseal canal engaging stems and metaphyseal cementation); c) Full uncemented (porous implants to provide ingrowth); d) Systems associated with metaphyseal cones.^10^, ^11^, ^12^, ^13^, ^14^, ^15^, ^16^, ^17^, ^18^, ^19^, ^20^, ^21^ There are three types of ligament constraint in RKA: a) Posterior stabilized (PS, Dished and medial pivot), used with the competent collateral ligaments; b) Non-linked Condylar Knee (CCK), used with partial collateral ligaments tear; c) Linked Rotating Hinge Knee (RHK), used with fully incompetent collateral ligaments.^1^^,^^3^^,^^6^^,^^7^^,^^10^ The last two have the disadvantage of impairing greater rotation stress to fixed interfaces and limiting Range of Motion (ROM).^1^^,^^2^^,^^6^^,^^7^

Brazil does not have a national arthroplasty registry, and very few published papers are available to study prostheses manufactured in this country.

The MBV revision knee (Metabio Industrial, Rio Claro, Brazil) is a modular system that can be implanted with full cemented and hybrid fixation, has PS and CCK liners and full interchangeability (one can combine any femoral with any tibial components). No previous studies have reported the results of using this implant.

The purpose of this retrospective study was to determine the clinical and radiographic outcomes of RKA using MBV prosthesis after a minimum of two years follow-up in 31 knees.

## Material and methods

Before starting data collection, the institutional review board approved this study (CAAE: 05424312.1.0000.0068). This study followed the CONSORT Statement rules.

Between November 2010 and January 2017, 31 RKAs were performed by the first author using the MB-V implant in 30 patients (one had bilateral). There were 19 women and 11 men, ranging from 55 to 83 years (mean 66 years) at the time of the surgery. The mean follow-up was 9.1 years (range: 2.3 to 13.3) (Table 1).

**Table 1:** Patients’ clinical data.

**Sex**	**Male**	**Female**
	11	19
**Age**	**Mean**	**Range**
	68	55‒83
**Side**	**Right**	**Left**
	14	17
**Follow-up (31 knees) (years)**	**Mean**	**Range**
	9.1	2.3‒13.3
**Reason for revision (31 knees)**	**Number**	**%**
Infection	15	50
Aseptic loosening	14	43.3
Arthrofibrosis	2	6.6

Reasons for revision were: a) Infection (n = 15); b) Arthrofibrosis with implant mal-position (n = 2); c) Aseptic loosening (n = 14: n = 8: all components were loose; n = 5: only the tibial component, n = 1 only the femoral component). A partial revision was performed in three knees (one femoral and two tibial components plus a PS liner), since the other component was compatible, fixed, and in a good position. In the remaining cases, all implants were exchanged (Table 1).

The infected knees were treated with a two-stage technique,^1^^,^^12^^,^^13^ and implantation of a static vancomycin-loaded cement spacer. Thirteen cases had a bacterial infection (*Staphylococcus aureus* n = 6; *Pseudomonas aeruginosa* n = 5; negative culture n = 2) that were treated with a 6-week course of intravenous antibiotics and reimplantation after three months. Two cases with fungal infection (*Candida sp.*) were treated with six months of oral fluconazole, and reimplantation after seven months. No antifungal agent was added to the spacer since the results of the cultures came after the prosthesis removal.

Before the surgery, all patients were evaluated with blood tests, CRP levels, and cardiological consultation. All knees were aspirated before the procedure. Bone defects were categorized by AORI classification^22^, ^23^, ^24^ preoperatively (X-Ray) and intra-operatively (after implant/spacer removal).

All knees were accessed by medial parapatellar arthrotomy. Prior implants were removed with care to preserve bone stock. Medullary canal was progressively reamed by hand until cylindrical endosteal contact of the diaphysis was achieved. The MBV instruments were used for proper femoral and tibial cuts. Pre-mixed cement with antibiotics was used in 29 knees. Amphotericin B was mixed with conventional cement in prior fungus infection cases.^25^

**Fig. 1:**
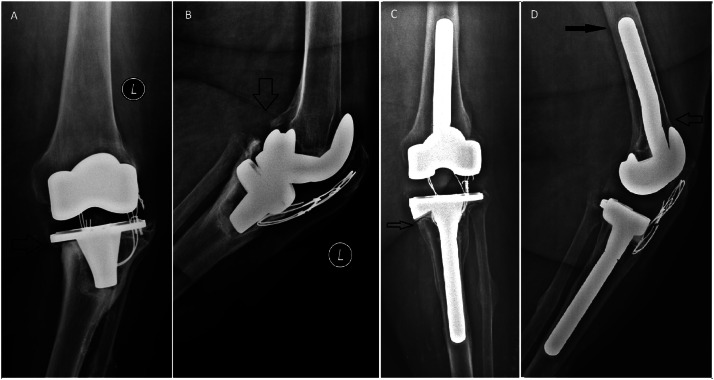
(A) Loose tibial component with varus migration (AORI 2A defect.). (B) Posterior femoral osteolysis. (C) Postoperative (9 years). The arrow shows radiolucent line beneath medial tibial 26° wedge. (D) Postoperative (9 years). Open arrow shows offset stem positioning the femoral component in full contact with the anterior cortex. Black arrow shows the pedestal sign at the tip of the stem. The cerclage wires were not removed to avoid complications to the extensor mechanism. The patient had no complaints after the surgery.

In fourteen tibias, offset stems were used to centralize the component to the metaphyseal bone.^26^ Seven knees had flexion contracture (FC) that were corrected by upsizing the femoral component plus an offset stem (Fig. 1A-C). No additional distal femoral bone cut was performed, preventing *patella baja* ^27^ (Fig. 1A-C). Metal Block/wedges were adopted in 17 femurs and 22 tibias. Additional cement/screw was used to fill larger defects when blocks were not sufficient, in three tibias and four femurs.^14^ No additional allogeneic bone grafting was performed (the institution is not registered with a bone bank).

Hybrid fixation was used in all 30 tibias and in 27 femurs. The length of the stem was preoperatively selected using long X-Ray, to achieve at least 4 cm cylindrical diaphyseal fixation.^17^ Two femoral components were fully cemented. The first case was initially categorized as 2A AORI^22^ femoral defect, but intraoperatively there was a large cavitary defect (AORI 3). The femoral component was unstable rotationally even with the use of augments and a long stem. The second case was a 2B femoral defect with sclerotic metaphyseal bone. The surgeon decided to fully cement the implant because it would be difficult to interdigitate the cement to the bone. Both components were fixed with a short 75 mm stem (2 mm smaller than the last reamer), bone plug, and PS liner.

Intra-operatively, valgus and varus stress tests were performed after final implants were cemented, to assess collateral ligament integrity with a trial PS liner. When the ligament integrity was doubtful or inadequate, the CCK liner was implanted (six knees).

Clinical assessments that involved a physical examination (emphasis on ROM and valgus/varus stability) were performed at every post-operative follow-up visit (at two, four and six weeks; six months, one year after the surgery, and then annually).^20^ To access function, the authors used KSS (Knee Society Score) and KSS-function (Knee Society Score – Function) scores at six months and annually.^24^

Long anteroposterior and lateral radiographs were obtained at routine follow-up visits. Those were assessed using the Knee Society Roentgenographic Evaluation System^22^ for lower limb alignment, component position (and possible migration/subsidence), presence of radiolucent lines (≥ 1 mm in width) and osteolysis.

The Mann-Whitney test was applied to compare numerical measurements between two groups (infection and aseptic loosening). The Wilcoxon test for related samples was applied to compare results between two-time points. The significance level adopted for statistical tests was 5 % and the program used was SAS System for Windows (Statistical Analysis System), version 9.4. SAS Institute Inc, 2002‒2012, Cary, NC, USA.

## Results

### Clinical and radiological data

Patients’ results data are summarized in Table 2. Mean pre and postoperative ROM of the 31 knees were 50° (range 0° to 135°) and 107° (range 40° to 135°), respectively. Mean flexion contraction angle (FCA) was 10° (range 0° to 35°) preoperatively and 3° (range 0° to 10°) postoperatively. Mean postoperative KSS and KSS-function scores,^20^ according to the Knee Society system were 82 (range 38‒98 points) and 79 (range 20‒100 points). Fourteen patients had tibial shin pain (located at the tip of the stem) that disappeared within two years.

**Table 2:** Patients clinical and function results.

	**Mean**	**Range**		**Mean**	**Range**
**Preoperative FCA**	10	0‒35	**Postoperative FCA**	3	0‒10
Infection	7.6	0‒20	Infection	2	0‒10
Loosening	13.6	5‒35	Loosening	4.2	0‒10
Arthrofibrosis	5	0‒10	Arthrofibrosis	5	0‒10
**Preoperative ROM**	50	0‒135	**Postoperative ROM**	107	40‒135
Infection	0	0	Infection	100	40‒135
Loosening	106.4	80‒135	Loosening	119.6	100‒135
Arthrofibrosis	32.5	25‒40	Arthrofibrosis	65	60‒70
**Postoperative KSS**	82	38‒92	**Postoperative KSS-f**	79	20‒100
Infection	80.6	38‒92	Infection	76.6	200‒100
Loosening	89.3	80‒92	Loosening	89.4	72‒100
Arthrofibrosis	41.5	40‒43	Arthrofibrosis	41.5	38‒45

Radiographic preoperative evaluation: 21 knees were in varus (mean 8°, range 1° to 20°), and 10 were in valgus (mean 3°, range 1° to 6°). Postoperatively: all knees were in valgus (mean 5°, range 3° to 7°). All tibial components were in 90°±1° but one, was implanted in a 3° varus (due to prior deformity and a narrow 10 mm canal). It was decided to accept this position to avoid an osteotomy.^28^ All femoral components were implanted in 6° valgus ±1°.

Bone defects were categorized (AORI classification)^22^ intraoperatively for the femur as type 1 (n = 11), 2A (n = 7), 2B (n = 10), and 3 (n = 1); for the tibia as type 1 (n = 10), 2A (n = 15), and 2B (n = 5). There was a good pre-and intraoperative evaluation agreement, with an error in two femurs (preoperatively both as 2A, but intra-operatively as 2B and 3).

The last follow-up X-Ray showed cement-bone radiolucency in 7/30 tibial- (in zones one and two, beneath the tibial plate) and 1/29 femoral components (the case with AORI 3). The sums of scores of radiolucent lines around tibial and femoral components, as determined using the Knee Society Roentgenographic evaluation system, were ≤4 for all knees. Pedestal sign at the tip of the stem was present in 26/30 tibias and 27/29 femurs (Fig. 1D).^29^ Pedestal signs did not correlate with pain or any complication.

**Fig. 2:**
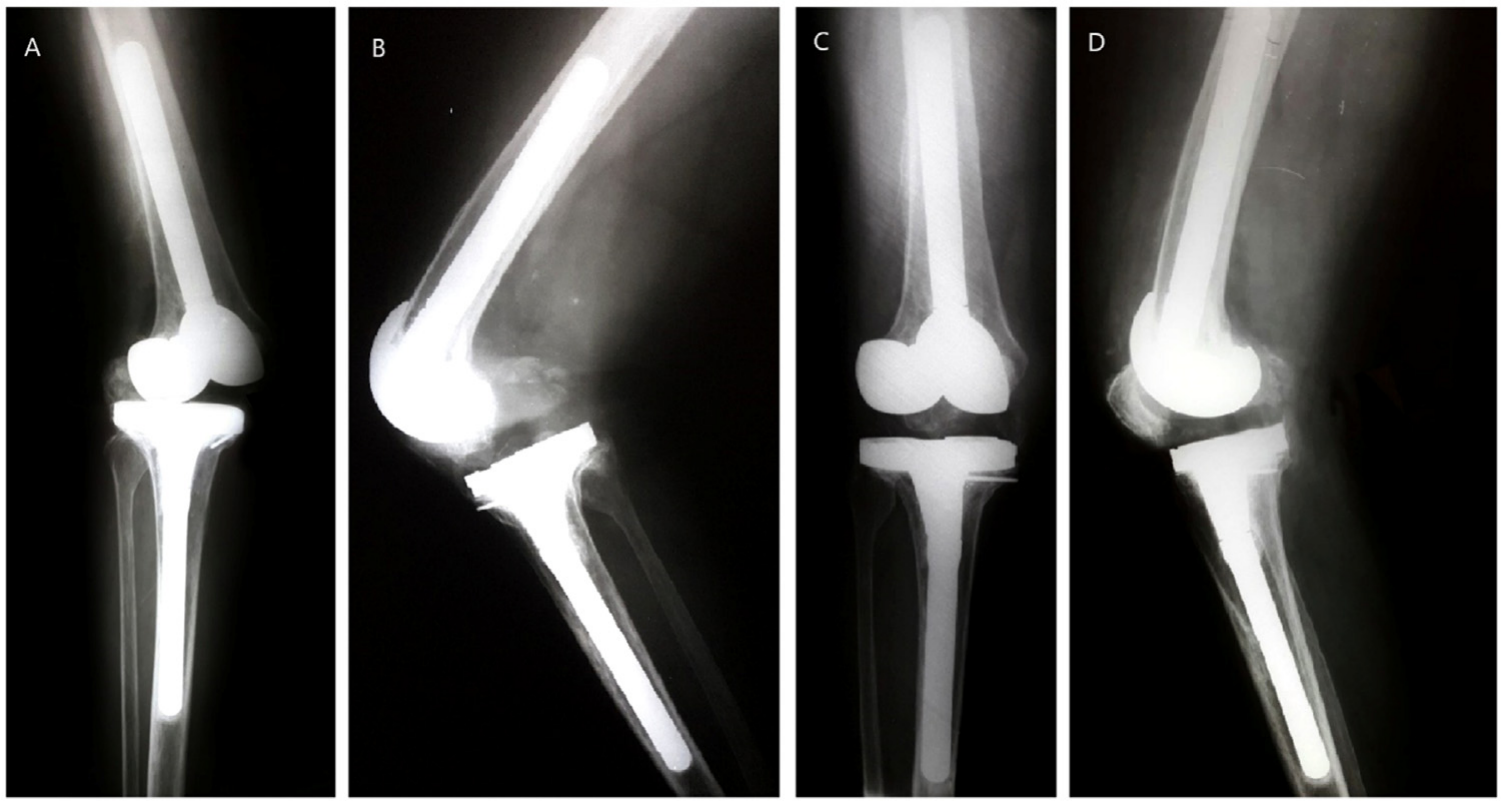
(A) Posterior dislocation. (B) Medial opening with avulsion of medial collateral ligament. (C) 6.1 years postoperative single leg standing AP X-Ray showing no medial opening. (D) Lateral view showing good position with patella baja.

The cases in which complications occurred were: a) The first patient (prior diagnosis of aseptic loosening) had an acute postoperative infection (*Klebsiella ESBL*) and was treated with Irrigation, Debridement, Liner Exchange (IDLE), and intravenous Ertapenem for six weeks, without recurrence after 12 years; b) The second patient (prior infected knee) had a hematogenous infection, initially treated with IDLE at 3.7 years post-operative. Since the infection could not be controlled, the prosthesis was removed and a static spacer loaded with vancomycin was implanted. During the surgery, the implants were fixed. It should be noted that he was satisfied with the outcome before the infection occurred; c) The third case was a prior fungus-infected knee that was revised using a PS insert. After three months, the patient sustained valgus torsion of the knee, with posterior dislocation and full medial collateral ligament avulsion. She was treated with closed reduction and a brace in full extension for 6 weeks, but the knee remained unstable. Five months after the surgery a polyethylene exchange was performed with a 3 mm thicker CCK liner and reconstruction of the medial collateral ligament through Krackow's technique.^30^ Three months after the patient had a stable knee, a ROM of 0° to 100°, and almost no pain. The patella became baja after the last surgery (Fig. 2A-D). After 7.2 years the knee became infected again, this time by a bacterium (*E coli*). The implants were removed, and a mobile spacer was implanted.

There were four poor results: a) The first patient (preoperative fungal infection) evolved with severe flexion instability after the surgery, even with the use of a CCK liner. She used a full-time walker and could not properly go up or down stairs. During the procedure, the CCK liner appeared to stabilize the knee, but it proved to be insufficient. This patient should have been operated with an RHK. After 2.3 years she had a cerebral stroke and died; b) One patient evolved with arthrofibrosis (pre-infected knee with a static spacer). After thirty days, the ROM was 0°‒40° and maintained so. Even with physiotherapy and manipulation, the knee remained stiff; c) Both patients with preoperative arthrofibrosis had implants in malposition (one with a femoral internal rotation, and the other with tibia in varus and in internal rotation). Preoperative ROM was 0°‒40° and 10°‒25°. At four weeks ROM was 0°‒100° and 10°‒90° but decreased to 0°‒70° and 10°‒60° after three months. KSS and KSS-function scores were 40/38 and 43/45 respectively. Both patients had a poor outcome, with continuous pain but felt better after the surgery.

There was no aseptic loosening or wear of the liner, with two cases failing due to infection (93.3 % survival in 31 knees). Twenty-six patients (27 knees, 87 %) were fully satisfied six months after the surgery. One patient regretted doing the surgery (the second case described as poor outcomes), stating that it felt better with the static spacer. Function scores were restored, with a mean KSS of 82 and KSS-function scores of 79. All patients but two had at least five years of follow-up (one died within 2.3 years and the other had a prosthesis removal at 3.6 years).

Comparing the infection group (n = 15) with the loosening group (n = 14), the authors found a statistical difference in the postoperative ROM and KSS with better results in the loosening group. Regarding the range of motion, the difference was expected, since the infected patients were using static cement spacer. KSS-Function was similar in both groups. The arthrofibrosis group was not analyzed due to its small number (n = 2) (Table 2, Table 3 and Table 2, Table 3).

**Table 3:** Descriptive analysis and comparisons between groups.

**Variable**	**Infection (n** = **15)**	**Loosening (n** = **14)**	**Total (n** = **29)**	**p-value**
**KSS ‒ Mean±SD**	80.60 ± 16.45	89.29 ± 3.20	84.79 ± 12.63	0.0445^a^
**KSS ‒ Median (min‒max)**	88.00 (38.00‒92.00)	89.50 (80.00‒92.00)	89.00 (38.00‒92.00)	
**KSS-f, Mean** ± **SD**	76.58 ± 24.21	89.43 ± 9.37	82.78 ± 19.40	0.1585^a^
**KSS-f, Median (min‒max)**	80.00 (20.00‒100.00)	90.00 (72.00‒100.00)	88.00 (20.00‒100.00)	
**ROM (Mean** ± **SD)**	100 ± 25	120 ± 9	110 ± 21	0.0071^a^
**ROM Median (min‒max)**	100 (40‒135)	120 (110‒135)	110 (40‒135)	

KSS, Knee Society Score; KSS-f, KSS-function; ROM, Postoperative Range of Motion.

*Arthrofibrosis group was excluded due to its small number of cases.

a Based on Mann-Whitney test.

## Discussion

RKA is a challenging surgical procedure, encompassing multiple corrections, such as articular deformity, bone loss, fixation in poor bone quality, joint stability, and range of motion. The RKA system and its instrumental must provide a solution to these problems.^1^, ^2^, ^3^, ^4^, ^5^, ^6^, ^7^, ^8^, ^9^, ^10^, ^11^, ^12^, ^13^, ^14^, ^15^, ^16^, ^17^^,^^26^^,^^27^

The correction of the mechanical axis in RKA is easily done with hybrid fixation in bones with no deformity.^28^ In the bowed or deformed canal, a short-cemented stem can be used and implanted in an angular position but it is less accurate.^28^ Both methods of fixation have shown equal results in terms of function and implant survivorship, in long-term studies.^11^, ^12^, ^13^, ^14^, ^15^, ^16^, ^17^ However, hybrid fixation facilitates the removal of implants and a new revision (since there is no cement in the canal).^13^ In hybrid fixation, there must be some metaphyseal bone support to the components (AORI defect maximum of 2B).^31^ In a former study, the authors obtained the same results.^31^

Bone loss can be treated by five methods: cement, screws plus cement, metal blocks/wedges, bone (autologous or homologous), and trabecular metal cones/sleeves.^10^^,^^18^, ^19^, ^20^, ^21^ Any of the first three methods can fill defects up to 2B. They are safe, easily done, of low cost, and have proven results.^10^^,^^31^^,^^32^ For the AORI type 3 femoral defect in this study, a full-cemented technique was performed with good fixation and functional outcomes after 7.6 years. However, there is a risk of fracture of the femoral component/stem junction, due to the lack of metaphyseal bone support.^12^

Both cases of postoperative instability had 2B bone loss, ligament scars, and prior fungal infection. In the first case, the reconstruction of the medial collateral ligament and insertion of a thicker CCK liner was enough to correct the instability. The second had severe flexion instability even with the use of the CCK liner. This liner should only be used with partial collateral ligament insufficiency and relatively balanced extension/flexion spaces.^6^^,^^7^^,^^10^

Pain at the end of the stem is frequent in hybrid fixation as shown by Barrack and Kimpton.^29^^,^^33^ It was present in 46.6 % of the tibias (14/30 knees) but in no femur. The pain was over within two years in all cases. No further treatment was necessary. Patients need to be warned about this before surgery. Pedestal signs were frequent and other authors have published comparable results. They found no correlation with symptoms or complications.^12^^,^^13^^,^^31^

Single component revision was performed in three knees, with good results and no complications. Rodrigues et al. obtained the same results. The cost is lower, and the surgery is less complex.^34^

The MBV revision system showed survivorship comparable to other series (93.5 % for all reasons and no aseptic loosening) in a medium of 9.1 years follow-up. Sheng et al. (2006) and Koskinen et al. (2008) reported, in the Finnish Arthroplasty Registry, a five-year survivorship rate of 89 % in 2637 RKA, operated between 1990 and 2002.^35^^,^^36^ Using other types of implants, Laskin et al. (58 knees), Meijer et al. (60 knees) and Wilke et al. (234 knees) obtained results similar to ours (5-year survivorship of 96 %, 92 %, and 91 %, respectively).^37^, ^38^, ^39^ Also, the mean postoperative KSS and KSS-function scores the authors obtained were comparable to those of other authors [77 and 82]. Haas et al. (57 patients), Lee et al. (27 patients), and Ye et al. (51 patients) got, respectively, 88/76; 88.5/72.4; and 85.3/85.^1^^,^^5^^,^^8^

## Conclusion

The MBV modular revision system showed 100 % survivorship to aseptic loosening and 93.5 % overall survivorship in a mean follow-up of 9.1 years. Twenty-six patients (27 knees, 87 %) were fully satisfied. This system provided a solution to complex scenarios, even in cases of major bone loss, resulting in good stability, increased function, and range of motion. However, this system should not be used in cases of major instability, since there are no RHK implants.

## Authors’ contributions

Francisco Fontes Cintra: Main author.

Mauricio Etchebehere: Helped the main author in many surgeries and collected data; revision of the information, helped to write the paper.

Eduardo Rached: Helped the main author in many surgeries and collected data.

Giancarlo Cavenaghi: Helped the main author in many surgeries and collected data.

Paulo Eduardo Dias Rahal: Helped the main author in many surgeries and collected data.

Rodrigo Gonçalves Pagnano: Helped the main author in many surgeries and collected information; Revision of the paper.

## Conflicts of interest

The authors declare no conflicts of interest.
